# Synthesis, Anti-Tumor and Anti-Angiogenic Activity Evaluations of Asiatic Acid Amino Acid Derivatives

**DOI:** 10.3390/molecules20047309

**Published:** 2015-04-21

**Authors:** Yue Jing, Gang Wang, Ying Ge, Minjie Xu, Zhunan Gong

**Affiliations:** Center for New Drug Research and Development, College of Life Science, Nanjing Normal University, Nanjing 210023, China; E-Mails: jingyueshadow@126.com (Y.J.); jiyehanyan@126.com (G.W.); emilygy0131@126.com (Y.G.); xmj1011@163.com (M.X.)

**Keywords:** asiatic acid, amino acid derivatives, anti-tumor activity, anti-angiogenic effect, stability, HPLC analysis

## Abstract

Fifteen semi-synthetic derivatives of asiatic acid (AA) have been synthesized and evaluated for their biological activities. The successful modification of these compounds at the C-2, C-3, C-23 and C-28 positions was confirmed using NMR, MS and IR spectra. Further, their anti-tumor effects were evaluated *in vitro* using different cancer cell lines (HeLa, HepG2, B16F10, SGC7901, A549, MCF7 and PC3), while their anti-angiogenic activities were evaluated *in vivo* using a larval zebrafish model. Among the derivatives, compounds **4**–**10** showed more potent cytotoxic and anti-angiogenic effects than AA, while compounds **11**–**17** had significantly less effects. The new derivative **10** was also included in finished formulations to evaluate its stability using HPLC due to its potential topical use. The derivative **10** had markedly better anti-tumor activities than both AA and other derivatives, with similar stability as its parent compound AA.

## 1. Introduction

**Figure 1:**
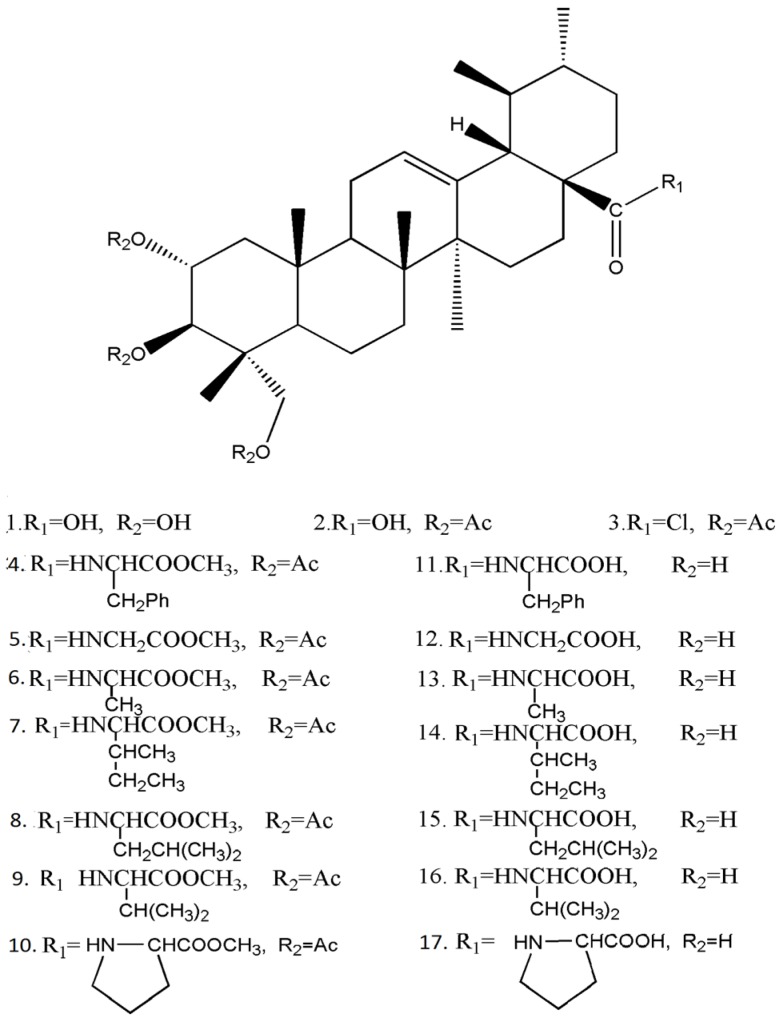
Structures of asiatic acid (**1**) and its derivatives **2**–**17** tested in the present study.

Asiatic acid (AA, 2α,3β,23-trihydroxyurs-12-ene-28-oic acid, Figure 1), one of the active pentacyclic triterpenoids found in *Centalla asiatica*, can be easily prepared from hydrolysis of asiaticoside. Besides its traditional usage to treat skin defects [1], AA also has other biological effects including anti-tumor [2,3,4,5,6], anti-inflammation [7], hepatoprotective [8], anti-depression, and anti-Alzheimer’s disease [9,10] activities, like other triterpenes.

However, the efficacy of the original AA is relatively poor. Many attempts have been made to improve this. For example, 2-hydroxypropyl-β-cyclodextrin has been used as an adjuvant for enhancing the encapsulation and releasing characteristics of asiaticoside [11]. Poly(l-lactide) (PLLA) nanoparticles loaded with asiatic acid (AA) have also been successfully produced using the rapid expansion of a subcritical solution into an aqueous receiving solution containing a dispersing agent [12]. Moreover, many researchers have synthesized various AA derivatives by adding new groups to AA [13,14,15]. Increasing the solubility of a compound usually can improve its bioavailability. For example, conjugation of an amino acid to oleanolic acid has been to shown to improve its water solubility as well as its anti-melanoma activity [16]. It is reported that a hydrogen donor group at either the C-3 position and/or C-28 positions of ursolic acid is essential for its cytotoxic activity [10]. To this end, a series of AA derivatives were synthesized by substituting seven different amio acids at positions of C-28. Their cytotoxic activities were then evaluated *in vitro* using seven cancer cell lines (HeLa, HepG2, B16F10, SGC7901, A549, MCF7 and PC3). We then sought to evaluate the anti-angiogenic activity of the derivatives using Tg(fli1:EGFP) zebrafish. Results showed that acetylation of the C-2, C-3, and C-23 hydroxy groups in conjunction with a substituted amino acid ester group at C-28 (compounds **4**–**10**), resulted in derivatives not only having stronger cell growth inhibitory activity, but also exhibiting more powerful anti-angiogenic effects than AA.

## 2. Results and Discussion

### 2.1. Chemistry

**Scheme 1:**
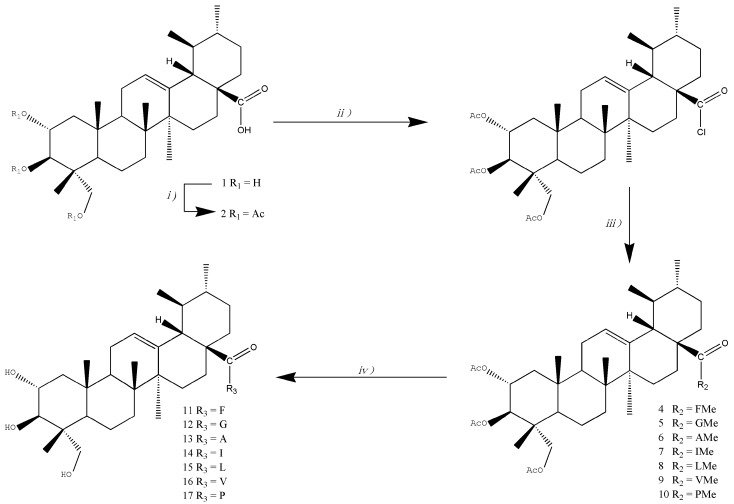
Synthesis of amino acid derivatives of asiatic acid.

As shown in Scheme 1. AA (**1**) was used as the starting material, and a series of amino acid derivatives were synthesized. Full acetylation of **1** afforded 2α,3β,23-O-triacetylasiatic acid (**2**) in good yield. Treatment of **2** with COCl_2_ afforded the corresponding acyl chloride **3**, which was used for the following reactions without further purification. Reaction of **3** with concentrated Et_3_N solution and amino acid methyl ester hydrochlorides furnished amides **4**–**10**, which were hydrolyzed with aqueous NaOH to give asiatic amides **11**–**17** (Scheme 1).

### 2.2. Antitumor Activity of the Compounds

Seven different kinds of cancer cell lines (HeLa, HepG2, B16F10, SGC7901, A549, MCF7 and PC3) were chosen to determine the cytotoxic activity of AA and its derivatives. The antiproliferative effects of the compounds were dertermined using Cell Counting Kit-8, in which WST-8(2-(2-methoxy-4-nitrophenyl)-3-(4-nitrophenyl)-5-(2,4-disulfophenyl)-2*H*-tetrazolium mono- sodium salt) was used as a substrate.

#### 2.2.1. IC_50_ Values of the Compounds.

As shown in Table 1, compounds **4**–**10** with ester functions were found to have lower IC_50_ values than those of compounds **11**–**17**, and also showed stronger anti-tumor activities than their parent compound AA. These results suggest that: (1) compounds with acetylated hydroxy groups at the C-2, C-3 and C-23 positions only showed less activities than AA; (2) compounds with only conjugated amino acids at C-28 also showed less activity than AA; (3) compounds with both acetylated hydroxy groups at C-2, C-3 and C-23 positions, and an amino acid ester group at C-28, had stronger activities than AA. Meanwhile, these activities varied based on alkyl side chains on the C-28 amide chain.

**Table 1:** Inhibitory effects of AA and derivatives on proliferation of A549, B16F10, Hela, HepG2, SGC7901, MCF7 and PC3 cells.

Compound	IC_50_ (µM)						
	A549	B16F10	Hela	HepG2	SGC7901	MCF7	PC3
**1**	18.8 ± 2.3	20.4 ± 2.9	55.1 ± 2.1	4.0 ± 1.4	36.8 ± 2.1	32.8 ± 0.4	53.6 ± 2.0
**2**	21.1 ± 4.5	>50.0	24.2 ± 1.6	10.2 ± 0.3	nt	nt	17.5 ± 0.3
**4**	5.7 ± 1.4	2.9 ± 0.5	9.2 ± 0.4	0.3 ± 0.1	14.2 ± 1.3	12.1 ± 1.0	10.9 ± 0.5
**5**	7.4 ± 0.8	5.8 ± 0.3	11.0 ± 1.6	1.8 ± 0.5	4.5 ± 0.2	5.6 ± 0.3	>10.0
**6**	3.1 ± 0.5	4.4 ± 0.4	5.1 ± 0.3	0.3 ± 0.1	9.2 ± 0.7	3.9 ± 0.7	10.1 ± 1.3
**7**	2.0 ± 0.2	17.1 ± 0.9	4.2 ± 0.2	1.7 ± 0.2	4.7 ± 0.6	>50.0	>10.0
**8**	33.9 ± 6.3	3.8 ± 0.3	32.3 ± 1.4	1.4 ± 0.1	nt	17.0 ± 0.3	10.2 ± 1.3
**9**	2.4 ± 0.2	11.3 ± 0.7	3.7 ± 0.1	4.1 ± 0.3	9.6 ± 1.0	12.8 ± 0.9	>10.0
**10**	2.4 ± 0.4	4.2 ± 0.2	4.8± 0.3	0.9 ± 0.3	4.6 ± 0.1	4.5 ± 0.1	9.2 ± 2.0
**11**	nt	nt	nt	14.0 ± 1.1	nt	nt	>50.0
**12**	30.2 ± 4.0	>50.0	nt	18.7 ± 1.6	nt	>50.0	>50.0
**13**	21.8 ± 1.7	>50.0	19.2 ± 3.9	20.5 ± 0.2	nt	17.1 ± 0.7	nt
**14**	25.4 ± 3.6	>50.0	13.8 ± 1.6	nt	nt	nt	nt
**15**	nt	nt	nt	16.5 ± 1.6	nt	nt	>50.0
**16**	25.0 ± 4.6	24.2 ± 0.6	nt	nt	nt	nt	nt
**17**	nt	nt	21.3 ± 5.1	nt	nt	nt	nt

nt = not tested.

#### 2.2.2. Cell Viability Suppression Activity of the Compounds

**Figure 2:**
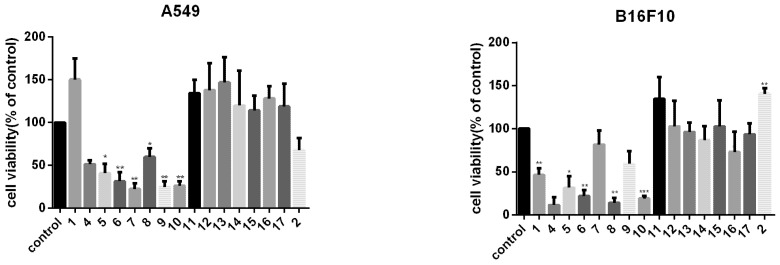

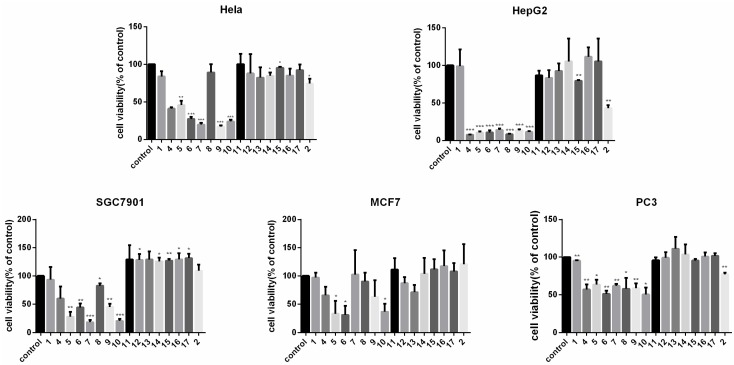
Cell viability suppression effect of AA derivatives on the A549, B16F10, HeLa, HepG2, SGC7901, MCF7, and PC3 cells. Cells were treated with 10 μM compounds for 72 h and the cell viability was measured. * *p* < 0.05, ** *p* < 0.01, *** *p* < 0.001.

To compare the anti-tumor effect of the derivatives with their parent compound AA, tumor cells were exposed to different compounds at 10 μM for 72 h. We found that compounds **4**–**10** showed a stronger cytotoxic effect on cell viability than AA, while compounds **11**–**17** showed a much smaller cytotoxic effect. Notably, AA-PMe (**10**) presented the strongest anti-tumor acitivity among all the compounds to most cancer cell lines (Figure 2).

### 2.3. Anti-Angiogenic Activity of the Compounds in Zebrafish

**Figure 3:**
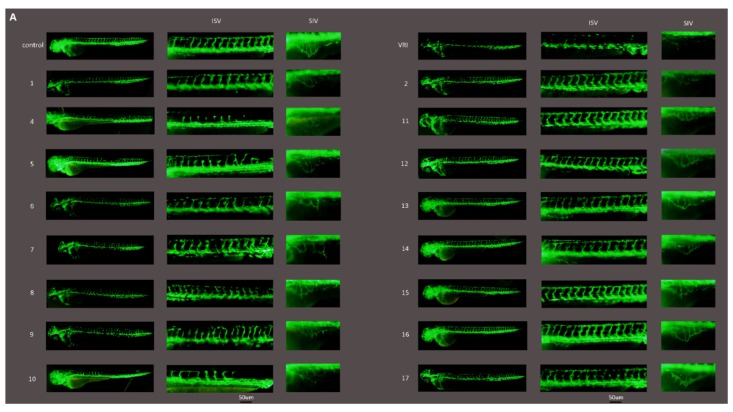

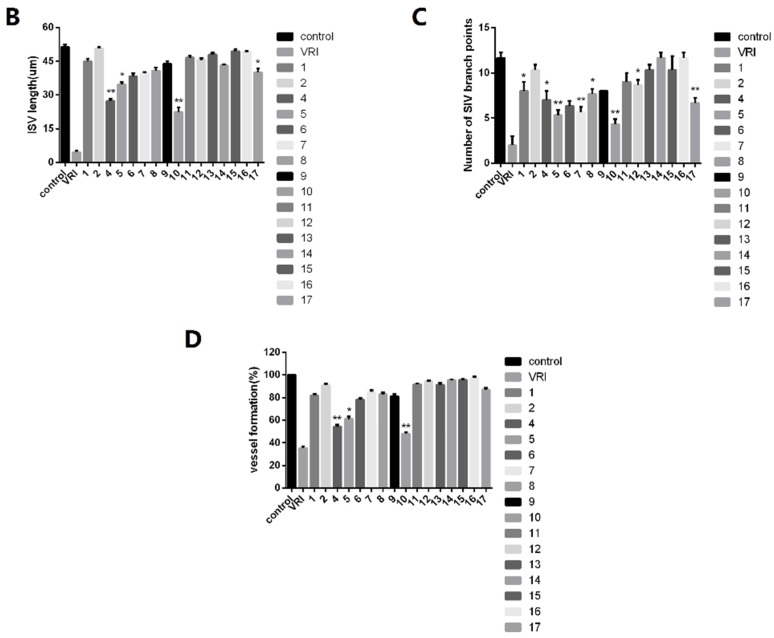
The anti-angiogenic activity of AA derivatives in zebrafish. Tg(fli1:eGFP) zebrafish embryos at 24 hpf were immersed in culture media containg 0.1% DMSO (control), 150 nM VRI (positive control) or 10 μM compounds. (**A**) Live fluorescence microscopy highlights EGFP expressing intersegmental blood vessels (ISVs) and the subintestinal vessel plexus (SIVs), and the later which appears as a smooth basket-like structure with 5–6 arcades. Scale bar, 50 μm. (**B**,**C**) Quantification of the ISV length and number of SIV branch points in 72 hpf zebrafish embryos in the vehicle control group and compounds treated groups. (**D**) Evaluation of the anti-angiogenic activity of the 16 compounds using EAP assay. * *p* < 0.05, ** *p* < 0.01.

The anti-angiogenic effect of AA and its derivatives was evaluated in Tg(fli1:eGFP) zebrafish by examining their effect on vessel formation in embryos. As shown in Figure 3A, the intersegmental blood vessels (ISVs) were the most easily observed angiogenic vessels in the embryos at 48 hpf. 

Compounds **4**–**10** showed an obvious inhibition on ISV formation at 10 μM, but no obvious effect was observed for compounds **11**–**17** at the same concentration. At 72 hpf, the subintestinal vessel plexus (SIVs) developed as a smooth basket-like structure with approximately 5–6 arcades in the vehicle control group. The numbers of SIV branch points in compounds **4**–**10** were much fewer than those in **11**–**17** treated groups. Among these compounds, compound **10** exhibited the strongest inhibition on vessel formation (Figure 3A–D). Compounds **5** and **10** were found to lead to pericardial edema (Figure 3A), which might because circulation was hampered by reduction in vessel formation [17].

### 2.4. Stability of the Compounds

**Figure 4:**
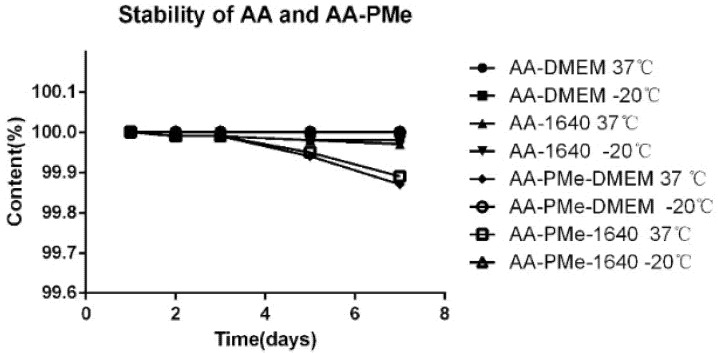
Stability of AA and AA-PMe (**10**) under different conditions.

The stability of AA and AA-PMe (**10**) was studied next. AA and AA-PMe (**10**) were kept in different media of DMEM and RPMI 1640 for several days and measured. Under all conditions, AA and AA-PMe (**10**) were soluble and stable without significant differences between different media, temperatures and time (Figure 4). We also found that both AA and AA-PMe (**10**) were stable at 37 °C and −20 °C. These results provided a theoretical basis for cell biology studies.

## 3. Experimental Section 

### 3.1. General

All reagents were obtained from Aladdin (Shanghai, China) and used without further purification. Thin-layer chromatography was shown with silica gel 60 GF_254_ (200–300 mesh). Infrared (IR) spectra were recorded by a Cary5000 instrument (Varian, Palo Alto, CA, USA). Nuclear magnetic resonance (^1^H-NMR) spectra were measured by an Avance 400 spectrometer (Bruker, Ettlingen, Germany) with DMSO-*d_6_* or CDCl_3_ as solvents and tetramethylsilane (TMS) as an internal standard. Chemical shifts (δ) were recorded in ppm, and coupling constant (*J*) in Hz. Mass spectra were recorded by a 1290/6460 LC-MS spectrometer (Agilent, Santa Clara, CA, USA). Melting points were determined using an RY-1 digital melting point apparatus (Baytree Packaging Machinery and Material Co., Limited, Shanghai, China). Asiatic acid was purchased from Guangxi Changzhou Natural Products Development Co., Ltd. (Nanning, China).

### 3.2. Synthesis and Characterization Data

Figure 1 shows the chemical structures of the AA derivatives, which were synthesized by modification of AA (**1**) at the C-2, C-3, C-23 and C-28 positions.

#### 3.2.1. Asiatic Acid (**1**) 

White solid; M.p. 230–231°; IR (KBr): 3382, 2919, 1689, 1047; ^1^H-NMR (DMSO-*d_6_*) δ 11.94 (brs, 1H, COOH), 5.14 (t, *J* = 3.3 Hz, 1H, H-12), 3.34–3.27 (m, 1H, H-2), 3.18 (d, *J* = 9.2 Hz, 1H, H-23), 3.04 (d, *J* = 8.5 Hz, 1H, H-23), 2.11 (d, *J* = 11.2 Hz, 1H, H-18), 1.05 (s, 3H), 0.93 (d, *J* = 5.6 Hz, 6H), 0.82 (d, *J* = 6.3 Hz, 3H), 0.74 (s, 3H), 0.54 (s, 3H); ^13^C-NMR (DMSO-*d_6_*) δ 178.75, 138.72, 125.00, 76.01 (C-3), 67.89 (C-2), 64.39 (C-23), 52.83, 47.42, 47.38, 47.26, 46.45, 42.94, 42.19, 39.56, 38.96, 38.90, 37.75, 36.78, 32.63, 30.66, 27.96, 24.27, 23.79, 23.44, 21.56, 17.90, 17.51, 17.43, 17.36, 14.23; ESI-MS: 489.7 ([M+H]^+^).

#### 3.2.2. 2α,3β,23-Triacetoxyurs-12-en-28-oic Acid (**2**)

Asiatic acid (**1**, 3 g, 6 mmol) was dissolved in pyridine (30 mL) and stirred for 0.5 h, then Ac_2_O (6.125 g, 60 mmol) was slowly added into the solution followed by about 1 h stirring and cooling. DMAP (0.03 g) was added to the mixture which was then stirred for 3 h at room temperature (RT). After slowly dropped and quickly stirred into ice to end the reaction, the mixture was extracted with CH_2_Cl_2_, and then acidified with aq. HCl. The org. layer was washed with sat. NaHCO_3_, and brine in sequence, dried (Na_2_SO_4_), and concentrated under reduced pressure. The crude product was purified by CC (petroleum ether(PE)/EtOAc 3:1) to give **2** (3.3 g, 92.8%). White solid; M.p. 93–95°; IR (KBr): 3380, 2920, 1740, 1230; ^1^H-NMR (DMSO-*d_6_*) δ 11.97 (s, 1H, COOH), 5.13 (t, *J* = 3.6 Hz, 1H, H-12), 5.09–5.01 (m, 1H, H-2), 4.94 (d, *J* = 10.3 Hz, 1H, H-3), 3.81 (d, *J* = 11.7 Hz, 1H, H-23), 3.50 (d, *J* = 11.8 Hz, 1H, H-23), 2.12 (d, *J* = 11.2 Hz, 1H, H-18), 2.01 (s, 3H), 1.98 (s, 3H), 1.92 (s, 3H), 1.05 (d, *J* = 7.7 Hz, 6H), 0.92 (s, 3H), 0.82 (d, *J* = 8.8 Hz, 6H), 0.76 (s, 3H); ^13^C-NMR (DMSO-*d_6_*) δ 178.69, 170.35 (COO), 170.17 (COO), 170.10 (COO), 138.71, 124.64, 74.72 (C-3), 69.50 (C-2), 65.36 (C-23), 52.83, 47.54, 47.31, 47.27, 43.68, 42.12, 41.98, 38.94, 38.91, 37.75, 36.73, 32.56, 30.66, 27.92, 24.23, 23.50, 23.36, 21.53, 21.15, 20.97, 20.94, 17.94, 17.39, 17.29, 17.03, 14.00; ESI-MS: 637.2 ([M+Na]^+^).

#### 3.2.3. *N*-(2α,3β,23-Acetoxyurs-12-en-28-oyl)acyl Chloride (**3**)

A mixture of **2** dissolved in CH_2_Cl_2_ and COCl_2_ was refluxed for 24 h at RT and the excess reagent was removed under reduced pressure. The residue was extracted with cyclohexane three times (50 mL each time) to give acyl chloride **3**.

#### 3.2.4. *N*-(2α,3β,23-Acetoxyurs-12-en-28-oyl)-l-phenylalanine Methyl Ester (**4**)

l-Phenylalanine methyl ester hydrochloride (6 mmol) in CH_2_Cl_2_ (200 mL) was added to **3**, and then Et_3_N was added (3 mL). The mixture was then stirred for 4 h at RT, washed with water, dried (Na_2_SO_4_), and concentrated under reduced pressure. The crude product was purified by CC (petroleum ether(PE)/EtOAc 3:1) to give **4** (2.7 g, 62%). White solid; M.p. 120–123°; IR (KBr): 3401, 2923, 1739, 1228; ^1^H-NMR (CDCl_3_) δ 6.35 (d, *J* = 6.2 Hz, 1H, N-H), 5.25 (t, *J* = 3.3 Hz, 1H, H-12), 5.19–5.10 (m, 1H, H-2), 5.07 (d, *J* = 10.3 Hz, 1H, H-3), 4.76–4.65 (m, 1H, H-2’), 3.84 (d, *J* = 11.8 Hz, 1H, H-23), 3.68 (s, 3H), 3.57 (d, *J* = 11.8 Hz, 1H, H-23), 2.08 (d, *J* = 10.9 Hz, 1H, H-18), 2.07 (s, 3H), 2.02 (s, 3H), 1.97 (s, 3H), 1.05 (d, *J* = 3.4 Hz, 6H), 0.94 (s, 3H), 0.87 (s, 3H), 0.83 (d, *J* = 6.4 Hz, 3H), 0.60 (s, 3H); ^13^C-NMR (CDCl_3_) δ 177.29, 172.13 (CON), 170.81 (COO), 170.42 (COO), 170.35 (COO), 138.36, 136.21 (Ar-C), 129.35 (Ar-C), 128.44 (Ar-C), 127.00 (Ar-C), 125.86, 74.77 (C-3), 69.88 (C-2), 65.22 (C-23), 53.53, 53.45, 52.14, 47.66, 47.56, 47.43, 43.72, 42.19, 41.88, 39.55, 39.02, 38.09, 37.71, 37.19, 32.27, 30.81, 27.64, 25.27, 24.68, 23.34, 23.22, 21.17, 21.07, 20.87, 20.78, 17.80, 17.07, 17.03, 16.41, 13.90; ESI-MS: 798.3 ([M+Na]^+^).

#### 3.2.5. *N*-(2α,3β,23-Acetoxyurs-12-en-28-oyl)glycine Methyl Ester (**5**)

As described for the preparation of **4**, treatment of **2** (5.8 mmol) with l-glycine methyl ester hydrochloride (6 mmol) afforded **5** (3.4 g, 85%). Yield: 85%; White solid; M.p. 174–177°; IR (KBr): 2921, 2865, 1741, 1367, 1228; ^1^H-NMR (DMSO-*d_6_*) δ 7.68 (t, *J* = 5.6 Hz, 1H, N-H), 5.18 (t, *J* = 3.5 Hz, 1H, H-12), 5.09–5.01 (m, 1H, H-2), 4.94 (d, *J* = 10.3 Hz, 1H, H-3), 3.79 (d, *J* = 17.0 Hz, 1H, H-2’) 3.64 (d, *J* = 11.6 Hz, 1H, H-23), 3.59 (s, 3H), 3.50 (d, *J* = 11.8 Hz, 1H, H-23), 2.16 (d, *J* = 10.9 Hz, 1H, H-18), 2.00 (s, 3H), 1.98 (s, 3H), 1.92 (s, 3H), 1.04 (d, *J* = 9.0 Hz, 6H), 0.93 (s, 3H), 0.83 (d, *J* = 3.5 Hz, 6H), 0.67 (s, 3H); ^13^C-NMR (DMSO-*d_6_*) δ 177.14, 170.93 (CON), 170.35 (COO), 170.17 (COO), 170.11 (COO), 138.68, 124.73, 74.74 (C-3), 69.51 (C-2), 65.37 (C-23), 52.30, 51.92, 47.56, 47.35, 46.98, 43.70, 42.03, 41.98, 41.35, 39.52, 39.19, 38.91, 37.73, 37.16, 32.57, 30.87, 27.66, 23.98, 23.51, 23.37, 21.58, 21.15, 20.97, 20.94, 17.93, 17.52, 17.03, 16.97, 14.00; ESI-MS: 686.3 ([M+H]^+^); 708.3 ([M+Na]^+^).

#### 3.2.6. *N*-(2α,3β,23-Acetoxyurs-12-en-28-oyl)-l-alanine Methyl Ester (**6**)

As described for the preparation of **4**, treatment of **2** (5.8 mmol) with l-alanine methyl ester hydrochloride (6 mmol) afforded **6** (2.4 g, 60%). Pale yellow solid. Pale yellow solid; M.p. 220–223°; IR (KBr): 2927, 2867, 1729, 1367, 1226; ^1^H-NMR (CDCl_3_) δ 6.57 (d, *J* = 5.9 Hz, 1H, N-H), 5.38 (t, *J* = 3.3 Hz, 1H, H-12), 5.20–5.10 (m, 1H, H-2), 5.07 (d, *J* = 10.3 Hz, 1H, H-3), 4.49–4.40 (m, 1H, H-2')3.83 (d, *J* = 11.8 Hz, 1H, H-23), 3.72 (s, 3H, OCH3), 3.56 (d, *J* = 11.8 Hz, 1H, H-23), 2.08 (d, *J* = 12.1 Hz, 1H, H-18), 2.07 (s, 3H), 2.01 (s, 3H), 1.97 (s, 3H), 1.35 (d, *J* = 7.0 Hz, 3H), 1.07 (s, 6H), 0.94 (s, 3H), 0.88–0.83 (m, 6H), 0.68 (s, 3H); ^13^C-NMR (CDCl_3_) δ 177.16, 173.71 (CON), 170.79 (COO), 170.41 (COO), 170.33 (COO), 138.51, 125.88, 74.76 (C-3), 69.87 (C-2), 65.22 (C-23), 53.50, 52.37, 48.13, 47.57, 47.45, 43.73, 42.26, 41.88, 39.61, 39.59, 38.97, 37.73, 37.25, 32.36, 30.81, 27.73, 24.59, 23.41, 23.22, 21.18, 21.07, 20.86, 20.76, 18.73, 17.82, 17.11, 17.05, 16.51, 13.89, 0.99; ESI-MS: 700.3 ([M+H]^+^); 722.3 ([M+Na]^+^).

#### 3.2.7. *N*-(2α,3β,23-Acetoxyurs-12-en-28-oyl)-l-isoleucine Methyl Ester (**7**)

As described for the preparation of **4**, treatment of **2** (5.8 mmol) with l-isoleucine methyl ester hydrochloride (6 mmol) afforded **7** (2.5 g, 58%). White solid. M.p. 206–208°; IR (KBr): 2962, 2873, 1739, 1363, 1226; ^1^H-NMR (CDCl_3_) δ 6.41 (d, *J* = 7.2 Hz, 1H, N-H), 5.37 (t, *J* = 3.2 Hz, 1H, H-12), 5.18–5.09 (m, 1H, H-2), 5.06 (d, *J* = 10.3 Hz, 1H, H-3), 4.50–4.45 (m, 1H, H-2'), 3.82 (d, *J* = 11.8 Hz, 1H, H-23), 3.68 (s, 3H, OCH3), 3.56 (d, *J* = 11.8 Hz, 1H, H-23), 2.07 (d, *J* = 12.0 Hz, 1H, H-18), 2.06 (s, 3H), 2.00 (s, 3H), 1.96 (s, 3H), 1.07 (s, 6H), 0.94 (s, 3H), 0.92 (d, *J* = 7.4 Hz, 3H), 0.89–0.85 (m, 6H), 0.83 (d, *J* = 6.9 Hz, 3H), 0.65 (s, 3H); ^13^C-NMR (CDCl_3_) δ 177.26, 172.38 (CON), 170.78 (COO), 170.41 (COO), 170.32 (COO), 138.42, 125.89, 74.75 (C-3), 69.87 (C-2), 65.21 (C-23), 56.15 (CN), 53.86, 51.87, 47.86, 47.58, 47.45, 43.72, 42.29, 41.87, 39.63, 39.60, 39.05, 38.42, 37.74, 37.59, 32.38, 30.82, 27.74, 25.52, 24.56, 23.39, 23.24, 21.14, 21.06, 20.86, 20.76, 17.82, 17.09, 17.03, 16.54, 15.14, 13.89, 11.53; ESI-MS: 742.4 ([M+H]^+^).

#### 3.2.8. *N*-(2α,3β,23-Acetoxyurs-12-en-28-oyl)-l-leucine Methyl Ester (**8**)

As described for the preparation of **4**, treatment of **2** (5.8 mmol) with l-leucine methyl ester hydrochloride (6 mmol) afforded **8** (2.7 g, 64%). White solid; M.p. 202–205°; IR (KBr): 2946, 2867, 1741, 1367, 1228; ^1^H-NMR (CDCl_3_) δ 6.31 (d, *J* = 7.1 Hz, 1H, N-H), 5.36 (t, *J* = 3.2 Hz, 1H, H-12), 5.18–5.09 (m, 1H, H-2), 5.06 (d, *J* = 10.3 Hz, 1H, H-3), 4.57–4.49 (m, 1H, H-2'), 3.82 (d, *J* = 11.8 Hz, 1H, H-23), 3.68 (s, 3H, OCH_3_), 3.56 (d, *J* = 11.8 Hz, 1H, H-23), 2.07 (d, *J* = 12.3 Hz, 1H, H-18), 2.06 (s, 3H), 2.00 (s, 3H), 1.96 (s, 3H), 1.07 (d, *J* = 3.4 Hz, 6H), 0.94 (s, 3H), 0.91 (d, *J* = 5.2 Hz, 6H), 0.86 (d, *J* = 5.2 Hz, 6H), 0.68 (s, 3H); ^13^C-NMR (CDCl_3_) δ 177.28, 173.55 (CON), 170.79 (COO), 170.41 (COO), 170.33 (COO), 138.45, 125.86, 74.76 (C-3), 69.87 (C-2), 65.22 (C-23), 53.67, 52.09, 50.74 (CN), 47.71, 47.59, 47.45, 43.73, 42.30, 41.87, 39.61, 39.58, 38.98, 37.74, 37.38, 32.40, 30.81, 27.71, 24.82, 24.63, 23.41, 23.20, 22.69, 22.41, 21.16, 21.06, 20.86, 20.76, 17.83, 17.10, 17.05, 16.62, 13.89; ESI-MS: 742.4 ([M+H]^+^); 764.4 ([M+Na]^+^).

#### 3.2.9. *N*-(2α,3β,23-Acetoxyurs-12-en-28-oyl)-l-valine Methyl Ester (**9**)

As described for the preparation of **4**, treatment of **2** (5.8 mmol) with l-valine methyl ester hydrochloride (6 mmol) afforded **9** (2.2 g, 54%). White solid. M.p. 205–208°; IR (KBr): 2937, 2871, 1735, 1367, 1228; ^1^H-NMR (CDCl_3_) δ 6.37 (d, *J* = 7.5 Hz, 1H, N-H), 5.37 (t, *J* = 3.3 Hz, 1H, H-12), 5.18–5.10 (m, 1H, H-2), 5.06 (d, *J* = 10.3 Hz, 1H, H-3), 4.46–4.39 (m, 1H, H-2'), 3.82 (d, *J* = 11.8 Hz, 1H, H-23), 3.69 (s, 3H, OCH3), 3.56 (d, *J* = 11.8 Hz, 1H, H-23), 2.08 (d, *J* = 12.2 Hz, 1H, H-18), 2.07 (s, 3H), 2.01 (s, 3H), 1.97 (s, 3H), 1.07 (s, 6H), 0.95 (s, 3H), 0.92–0.86 (m, 12H), 0.66 (d, *J* = 6.8 Hz, 3H); ^13^C-NMR (CDCl_3_) δ 177.47, 172.46 (CON), 170.79 (COO), 170.41 (COO), 170.33 (COO), 138.39, 125.91, 74.75 (C-3), 69.88 (C-2), 65.22 (C-23), 57.08, 53.89, 51.91, 47.96, 47.58, 47.46, 43.72, 42.29, 41.87, 39.63, 39.60, 39.07, 37.75, 37.71, 32.39, 32.00, 30.83, 27.74, 24.54, 23.40, 23.26, 21.14, 21.07, 20.86, 20.76, 18.70, 18.30, 17.82, 17.10, 17.03, 16.57, 13.89; ESI-MS: 750.4 ([M+Na]^+^).

#### 3.2.10. *N*-(2α,3β,23-Acetoxyurs-12-en-28-oyl)-l-proline Methyl Ester (**10**)

As described for the preparation of **4**, treatment of **2** (5.8 mmol) with l-proline methyl ester hydrochloride (6 mmol) afforded **10** (2.3 g, 62%).White solid. M.p. 180–181°; IR (KBr): 2921, 2865, 1743, 1367, 1228; ^1^H-NMR (DMSO-*d_6_*) δ 5.10–5.01 (m, 2H, H-12, H-2), 4.94 (d, *J* = 10.3 Hz, 1H, H-3), 3.80 (d, *J* = 11.6 Hz,1H, H-23), 3.56 (s, 3H, OCH3), 3.51 (d, *J* = 11.8 Hz, 1H, H-23), 2.31 (d, *J* = 11.1 Hz, 1H, H-18), 2.01 (s, 3H), 1.98 (s, 3H), 1.92 (s, 3H), 1.04 (d, *J* = 6.3 Hz, 6H), 0.92 (s, 3H), 0.85–0.81 (m, 6H), 0.68 (s, 3H); ^13^C-NMR (DMSO-*d_6_*) δ 174.46, 173.12 (CON), 170.37 (COO), 170.16 (COO), 170.10 (COO), 139.01, 124.45, 74.73 (C-3), 69.53 (C-2), 65.40 (C-23), 61.42, 51.83, 47.84, 47.63, 47.37, 43.67, 42.30, 41.97, 39.40, 38.85, 38.78, 37.79, 32.58, 31.79, 30.43, 27.79, 27.29, 25.90, 23.72, 23.34, 22.58, 21.54, 21.15, 20.99, 20.94, 17.86, 17.67, 17.04, 16.68, 14.01; ESI-MS: 726.3 ([M+H]^+^).

#### 3.2.11. *N*-(2α,3β,23-Hydroxyurs-12-en-28-oyl)-l-phenylalanine (**11**)

To a soln. of **4** (1 g, 1.6 mmol) in MeOH (20 mL) and THF (30 mL), 4 M NaOH (15 mL) was added dropwise, and the resulting mixture was stirred at RT for 2 h. The mixture was acidified with aq. HCl (4 M) to pH 3, added into ice water, and filtered. The crude product was purified by CC (petroleum ether(PE)/EtOAc 3:1) to give **11** in 68% yield. White solid; M.p. 211–214°; IR (KBr): 3376, 2915, 1627, 1045; ^1^H-NMR (DMSO-*d_6_*) δ 12.70 (s, 1H, COOH), 7.17 (d, *J* = 4.2 Hz, 1H, N-H), 5.06 (t, *J* = 3.2 Hz, 1H, H-12), 4.28–4.19 (m, 1H, H-2'), 3.51–3.41 (m, 1H, H-2), 3.30 (d, *J* = 10.7 Hz, 1H, H-3), 3.15 (d, *J* = 9.4 Hz, 1H, H-23), 3.04 (d, *J* = 10.2 Hz, 1H, H-23), 1.98 (d, *J* = 10.9 Hz, 1H, H-18), 0.96 (s, 3H), 0.90 (s, 3H), 0.84 (s, 3H), 0.79 (d, *J* = 6.3 Hz, 3H), 0.53 (s, 3H), 0.23 (s, 3H); ^13^C-NMR (DMSO-*d_6_*) δ 176.64, 173.76 (CON), 138.55, 138.52 (Ar-C), 129.77 (Ar-C), 128.48 (Ar-C), 126.65 (Ar-C), 125.16, 76.03 (C-3), 67.88 (C-2), 64.41 (C-23), 55.36, 54.64, 52.60, 47.47, 47.43, 46.89, 46.43, 42.92, 42.03, 39.09, 38.92, 37.65, 36.80, 32.24, 30.82, 27.61, 23.97, 23.76, 23.38, 21.60, 17.68, 17.61, 17.33, 16.36, 14.24; ESI-MS: 636.3 ([M+H]^+^); 658.3 ([M+Na]^+^).

#### 3.2.12. *N*-(2α,3β,23-Hydroxyurs-12-en-28-oyl)glycine (**12**)

As described for the preparation of **11**, compound **12** was obtained in 86% yield (0.68 g). White solid; M.p. 267–270°; IR (KBr): 3407, 2952, 1608, 1058; ^1^H-NMR (DMSO-*d_6_*) δ 12.40 (s, 1H, COOH), 7.45 (t, *J* = 5.2 Hz, 1H, N-H), 5.21 (t, *J* = 3.4 Hz, 1H, H-12), 3.72 (d, *J* = 17.4 Hz, 1H, H-2'), 3.57 (d, *J* = 17.4 Hz, 1H, H-2'), 3.52–3.44 (m, 1H, H-2), 3.30 (d, *J* = 10.5 Hz, 1H, H-3), 3.17 (d, *J* = 9.3 Hz, 1H, H-23), 3.04 (d, *J* = 10.4 Hz, 1H, H-23), 2.13 (d, *J* = 10.8 Hz, 1H, H-18), 1.04 (s, 3H), 0.91 (s, 6H), 0.84 (d, *J* = 6.3 Hz, 3H), 0.65 (s, 3H), 0.54 (s, 3H); ^13^C-NMR (DMSO-*d_6_*) δ 177.01, 171.88 (CON), 138.71, 125.25, 76.03 (C-3), 67.90 (C-2), 64.40 (C-23), 52.45, 47.48, 47.40, 46.95, 46.46, 42.94, 42.10, 41.36, 39.56, 39.23, 38.94, 37.72, 37.20, 32.58, 30.86, 27.71, 24.12, 23.80, 23.45, 21.62, 17.86, 17.63, 17.34, 17.01, 14.22; ESI-MS: 546.3 ([M+H]^+^); 568.3 ([M+Na]^+^).

#### 3.2.13. *N*-(2α,3β,23-Hydroxyurs-12-en-28-oyl)-l-alanine (**13**)

As described for the preparation of **11**, compound **13** was obtained in 78% yield. White solid; M.p. 227–231°; IR (KBr): 3382, 2921, 1629, 1047; ^1^H-NMR (DMSO-*d_6_*) δ12.42 (s, 1H, COOH), 7.27 (d, *J* = 6.7 Hz, 1H, N-H), 5.21 (t, *J* = 3.2 Hz, 1H, H-12), 4.20–4.07 (m, 1H, H-2'), 3.57 – 3.38 (m, 1H, H-2), 3.30 (d, *J* = 10.7 Hz, 1H, H-3), 3.17 (d, *J* = 9.4 Hz, 1H, H-23), 3.03 (d, *J* = 10.4 Hz, 1H, H-23), 2.11 (d, *J* = 10.8 Hz, 1H, H-18), 1.23 (d, *J* = 7.1 Hz, 3H), 1.04 (s, 3H), 0.91 (s, 6H), 0.84 (d, *J* = 6.3 Hz, 3H), 0.66 (s, 3H), 0.53 (s, 3H);^13^C-NMR (DMSO-*d_6_*) δ 176.26, 174.68 (CON), 138.52, 125.50, 76.02 (C-3), 67.90 (C-2), 64.39 (C-23), 52.69, 47.93, 47.48, 46.90, 46.46, 42.93, 42.16, 39.61, 39.17, 38.85, 37.71, 37.09, 32.69, 30.84, 27.75, 23.90, 23.77, 23.45, 21.59, 18.02, 17.85, 17.59, 17.36, 17.04, 14.23; ESI-MS: 560.4 ([M+H]^+^); 582.3 ([M+Na]^+^).

#### 3.2.14. *N*-(2α,3β,23-Hydroxyurs-12-en-28-oyl)-l-isoleucine (**14**)

As described for the preparation of **11**, compound **14** was obtained in 66% yield. White solid; M.p. 210–213°; IR (KBr): 3382, 2919, 1629, 1047; 1H-NMR (DMSO-*d_6_*) δ 12.45 (s, 1H, COOH), 6.92 (d, *J* = 7.3 Hz, 1H, N-H), 5.24 (t, *J* = 3.4 Hz, 1H, H-12), 4.16–3.96 (m, 1H, H-2'), 3.54 – 3.41 (m, 1H, H-2), 3.30 (d, *J* = 10.7 Hz, 1H, H-3), 3.17 (d, *J* = 9.3 Hz, 1H, H-23), 3.03 (d, *J* = 10.5 Hz, 1H, H-23), 2.08 (d, *J* = 10.6 Hz, 1H, H-18), 1.04 (s, 3H), 0.91 (d, *J* = 4.2 Hz, 6H), 0.84 (m, 6H), 0.81 (s, 3H), 0.79 (s, 3H), 0.63 (s, 3H), 0.53 (s, 3H). ^13^C-NMR (DMSO-*d_6_*) δ 176.52, 173.42 (CON), 138.28, 125.81, 76.02 (C-3), 67.87 (C-2), 64.37 (C-23), 56.71 (CN), 52.95, 47.51, 47.44, 47.33, 46.43, 42.94, 42.23, 39.58, 38.83, 37.69, 37.32, 37.15, 32.73, 30.88, 27.77, 25.52, 24.14, 23.73, 23.48, 21.54, 17.86, 17.54, 17.41, 17.32, 17.09, 15.75, 14.22, 11.69. ESI-MS: 602.3 ([M+H]^+^); 624.3 ([M+Na]^+^).

#### 3.2.15. *N*-(2α,3β,23-Hydroxyurs-12-en-28-oyl)-l-leucine (**15**)

As described for the preparation of **11**, compound **15** was obtained in 75% yield. White solid; M.p. 168–172°; IR (KBr): 3376, 2925, 1629, 1045; ^1^H-NMR (DMSO-*d_6_*) δ 12.33 (s, 1H, COOH), 7.23 (d, *J* = 7.7 Hz, 1H, N-H), 5.18 (t, *J* = 3.6 Hz, 1H, H-12), 4.20–4.08 (m, 1H, H-2’), 3.54–3.44 (m, 1H, H-2), 3.30 (d, *J* = 10.4 Hz, 1H, H-3), 3.17 (d, *J* = 9.4 Hz, 1H, H-23), 3.03 (d, *J* = 10.2 Hz, 1H, H-23), 2.16 (d, *J* = 10.9 Hz, 1H, H-18), 1.04 (d, *J* = 4.1 Hz, 3H), 0.91 (s, 6H), 0.87 (d, *J* = 6.3 Hz, 3H), 0.83 (s, 3H), 0.82 (d, *J* = 13.0 Hz, 3H), 0.66 (s, 3H), 0.54 (s, 3H); ^13^C-NMR (DMSO-*d_6_*) δ 176.77, 174.79 (CON), 138.47, 125.27, 76.02 (C-3), 67.90 (C-2), 64.39 (C-23), 52.54, 50.74 (CN), 47.50, 47.46, 47.04, 46.45, 42.93, 42.25, 39.18, 38.89, 38.81, 37.72, 36.99, 32.90, 30.89, 27.74, 24.74, 23.85, 23.78, 23.69, 23.55, 23.46, 21.82, 21.61, 17.89, 17.60, 17.50, 17.39, 14.23; ESI-MS: 624.3 ([M+Na]^+^).

#### 3.2.16. *N*-(2α,3β,23-Hydroxyurs-12-en-28-oyl)-l-valine (**16**)

As described for the preparation of **11**, **16** was obtained in 80% yield. White solid; M.p. 212–216°; IR (KBr): 3388, 2931, 1625, 1045; ^1^H-NMR (DMSO-*d_6_*) δ 12.65 (s, 1H, COOH), 6.89 (d, *J* = 7.3 Hz, 1H, N-H), 5.24 (s, 1H, H-12), 4.10–3.96 (m, 1H, H-2'), 3.55–3.42 (m, 1H, H-2), 3.30 (d, *J* = 9.6 Hz, 1H, H-3), 3.17 (d, *J* = 9.1 Hz, 1H, H-23), 3.03 (d, *J* = 9.6 Hz, 1H, H-23), 2.10 (d, *J* = 10.4 Hz, 1H, H-18), 1.04 (s, 3H), 0.94–0.82 (m, 15H), 0.63 (s, 3H), 0.53 (s, 3H); ^13^C-NMR (DMSO-*d_6_*) δ 176.80, 173.44 (CON), 138.37, 125.73, 76.00 (C-3), 67.90 (C-2), 64.37 (C-23), 57.74, 52.88, 47.50, 47.46, 47.39, 46.42, 42.93, 42.23, 39.59, 38.79, 37.69, 37.30, 32.73, 30.85, 27.76, 24.04, 23.75, 23.46, 21.54, 19.48, 19.10, 17.86, 17.53, 17.39, 17.13, 14.23; ESI-MS: 588.3 ([M+H]^+^).

#### 3.2.17. *N*-(2α,3β,23-Hydroxyurs-12-en-28-oyl)-l-proline (**17**)

As described for the preparation of **11**, compound **17** was obtained in 72% yield. White solid; M.p. 190–192°; IR (KBr): 3409, 2919, 1602, 1047; ^1^H-NMR (DMSO-*d_6_*) δ 12.09 (s, 1H, COOH), 5.05 (s, 1H, H-12), 4.21 (m, 2H, H-2’), 3.57–3.46 (m, 1H, H-2), 3.31 (d, *J* = 6.9 Hz, 1H, H-3), 3.17 (d, *J* = 9.3 Hz, 1H, H-23), 3.03 (d, *J* = 7.7 Hz, 1H, H-23), 2.32 (d, *J* = 11.0 Hz, 1H, H-18), 1.04 (s, 3H), 0.92 (s, 6H), 0.84 (d, *J* = 6.2 Hz, 3H), 0.66 (s, 3H), 0.54 (s, 3H);^13^C-NMR (DMSO-*d_6_*) δ 174.27, 174.17 (CON), 139.10, 124.74, 76.02 (C-3), 67.88 (C-2), 64.37 (C-23), 61.53, 55.37, 54.31, 47.83, 47.65, 47.50, 47.38, 46.55, 42.94, 42.41, 39.44, 38.88, 37.78, 33.28, 32.71, 30.44, 27.87, 27.44, 25.85, 23.99, 23.43, 22.62, 21.59, 17.83, 17.38,16.82, 14.23;ESI-MS: 586.3 ([M+H]^+^); 608.2 ([M+Na]^+^).

### 3.3. Cell Culure

HeLa, HepG2, B16F10, SGC7901, A549, MCF7 and PC3 cells were routinely maintained in Dulbecco’s modified eagle medium (DMEM), and B16F10 cells were cultured in RPMI 1640, supplemented with 10% fetal calf serum (Life Technologies, Carlsbad, California, USA) at 37 °C in humidified atmosphere of 5% CO_2_.

### 3.4. Antitumor Activity Assays

Cell proliferation and cytotoxicity of AA derivatives were determined using a Cell Counting Kit-8, in which WST-8(2-(2-methoxy-4-nitrophenyl)-3-(4-nitrophenyl)-5-(2, 4-disulfophenyl)-2H-tetra-zoli-um monosodium salt) was used as a substrate. Briefly, 1~5 × 10^4^ tumor cells were seeded in 96-well plates (200 μL per well) and cultured for 24 h, and then AA derivatives were added at different concentrations and incubated for 72 h. 10μL CCK-8 was added and the cells were incubated for another 1 h. The absorbance was measured at 450 nm with an ELISA reader (Thermo FC, Carlsbad, California, USA. Three independent experiments were carried out. The IC_50_ represents the drug concentration resulting in 50% growth inhibition. 

### 3.5. Embryo Handling

Transgenic zebrafish Tg(fli1:eGFP) with enhanced green fluorescent protein (EGFP) expressed in endotheliad cells(ECs) and wild-type zebrafish (Tuebingen line) were obtained from Model Animal Research Center of Nanjing University. Adult zebrafish were maintained at 28.5 °C and pH at 7 ± 0.2 in 14:10 h light/dark photoperiod, and fed with live brine shrimp once daily and dry food twice a day [18]. Embryos were produced by pairwise mating in fish hatch box. Embryos were maintained at 28.5 °C in E3 embryos medium (containing antifungal solvent [0.01% methylene blue]) and salts [5 mM NaCl, 0.17 mM KCl, 0.33 mM CaCl2, 0.33 mM MgSO4]) [19]. Normally developed embryos were dechorionated using forceps before drug treatment. All zebrafish studies were approved by the Institutional Animal Care and Use Committee at Nanjing University of Technology.

### 3.6. Assessment of Vessel Changes in Zebrafish Embryos by Fluorescent Microscopy

After drug treatment, zebrafish embryos were anesthetized with 0.016% tricaine (Sigma-Aldrich, Milan, Italy). The intersegmental blood vessels (ISVs) and subintestinal vessel plexus (SIVs) of embryos were observed and imaged at 48 and 72 hpf respectively under a fluorescence microscope (IX71, Olympus, Tokyo, Japan).

### 3.7. Quantitation of Endogenous Alkaline Phosphatase EAP in Zebrafish Embryo

During zebrafish development, the stage between 24 and 72 hpf has the highest angiogenic activity and the quantitative EAP assay was performed as described [20]. Tg(fli1:eGFP) transgenic zebrafish embryos (24 hpf) were arrayed in 96-well plate, one embryo per well, and incubated with embryo water (100 μL per well) containing AA and AA-PMe at a series of concentrations at 28.5 °C for continuously 48 h. In all experiments, 0.1% dimethyl sulfoxide (DMSO) was added as a carrier, and vehicle control with 0.1% DMSO was performed. Drug-treated embryos at 72 hpf were treated with increasing concentrations of ethanol for dehydration purpose.

Then the embryos were washed three times with diethanolamine buffer (Pierce, Rockford, Illinois, USA). Next, the embryos were stained according to the protocol described in phosphatase substrate kit. After staining, 50 μL 2 M NaOH was added to stop the reaction. The optical density of soluble end product was measured at 405 nm using a microplate reader. Vessel growth was presented as percentage in optical density compared with control [% vessel formation = (OD treated days 3 − OD control day 1)/(OD control day 3 − OD control day 1) × 100%]. Each assay was repeated at least three times.

### 3.8. Stability Studies

For further cytobiology research, we evaluated the behaviour of AA and AA-PMe (**10**) by performing a stability study in different media at different temperatures for different time.

#### 3.8.1. Solution Preparation

The samples were prepared using AA and **10** in different media. AA and **10** were solubilized in DMEM and RPMI 1640 medium and were divided into two temperature groups of 37 °C and −20 °C.

#### 3.8.2. HPLC Methods

AA: The determination was carried out under isocratic conditions, using acetonitrile (47.5)/water (52.5). Separation was monitored by absorbance detection at 215 nm. The flow rate was 1.0 mL/min, the injection volume was 20 μL and the separation process was performed at 35 °C.

AA-PMe (**10**): The determination was carried out under isocratic conditions, using acetonitrile (75)/water (25). Separation was monitored by absorbance detection at 215 nm. The flow rate was 1.0 mL/min, the injection volume was 20 μL and all the separation process was performed at 35 °C.

### 3.9. Statistic Analysis

All experiments were performed at least three times. Results were expressed as mean ± S.D., and all statistical comparisons were made by means of a one-way ANOVA test followed by Dunett’s t-test. *p* value less than 0.05 was considered statistically significant.

## 4. Conclusions 

In conclusion, 15 AA derivatives with modifications of the functional groups at C-2, C-3, C-23 and C-28 were synthetized. Their antitumor activities were evaluated *in vitro* using seven cancer cell lines, and their anti-angiogenc activities were evaluated using an *in vivo* larval zabrafish model. Results showed that most of AA derivatives with amino acid ester substitutions at C-28 such as compounds **4**–**10** had stronger cytotoxicity than AA. Also compounds with acetylated OH groups at C-2, C-3 and C-23, (compounds **4**–**10**) also showed stronger antitumor activities than compounds **11**–**17**. Evaluation of anti-angiogenic activities showed that compounds **4**–**10** also exhibited higher inhibition of angiogenesis than AA and other compounds in zebrafish. These results suggest that amino acid ester group-substitution at C-28 and acetylation of the OH at C-2, C-3 and C-23 might be important to their biological activities.
